# Testosterone elevation in ovarian adult granulosa cell tumor: A case report and review of the literature

**DOI:** 10.1097/MD.0000000000033763

**Published:** 2023-05-12

**Authors:** Ziwei Jiang, Yixuan Qiu, Siwen Hu, Yujing Li, Xing Chen, Yichao Jin, Huihua Dai

**Affiliations:** a Department of Gynecology, the First Affiliated Hospital with Nanjing Medical University, Nanjing, China.

**Keywords:** adolescent females, adult granulosa cell tumors, amenorrhea, hyperandrogenemia

## Abstract

Adult granulosa cell tumors (AGCT) mainly secret estrogen, but few androgens. It is rarer to have amenorrhea and hyperandrogenemia as clinical features. Here, we report a rare case of right side AGCTs with amenorrhea and hyperandrogenemia in a 19-year-old female.

**Patient concerns:**

The 19-year-old patient was admitted to our hospital due to amenorrhea for more than 1 year, and discovery of pelvic mass for 4 months. The gynecological ultrasound and computed tomography (CT) cannot define the nature of the mass. Surprisingly, an elevation in testosterone levels was also measured.

**Diagnosis and interventions:**

The present patient underwent laparoscopic right salpingo-oophorectomy and partial omentectomy and biopsy of the peritoneum.

**Outcomes:**

After the surgery, the testosterone value was down to normal. The patient menstrual cramps on August 13, 2021. Her clitoris is smaller than the front. Up to August 1, 2022, there was no obvious sign of recurrence.

**Lessons:**

Androgen-secreting AGCT is rare. We hope that this case can strengthen gynecologists’ early diagnosis and treatment of this disease and improve the prognosis.

## 1. Introduction

Ovarian granulosa cell tumors (GCT) originate from the ovarian sex cords. It is a common ovarian lock stromal tumor and is low-grade malignant accounting for 70% of all sex-cord tumors^[1]^ and 5% and 8% of all ovarian tumors,^[2]^ due to their clinical behavior and histological differences, these tumors are subdivided into 2 types: adult granulosa cell tumor (AGCT) and juvenile granulosa cell tumor. Among them, androgen-secreting granulosa cell tumors are rare, accounting for <3% of AGCTs,^[3]^ and those with signs of amenorrhea and virilization are even rarer. This article reports a case of AGCT mainly manifested as hyperandrogenemia and secondary amenorrhea and combined with literature review, the report is as follows.

## 2. Case presentation

**Figure 1.**
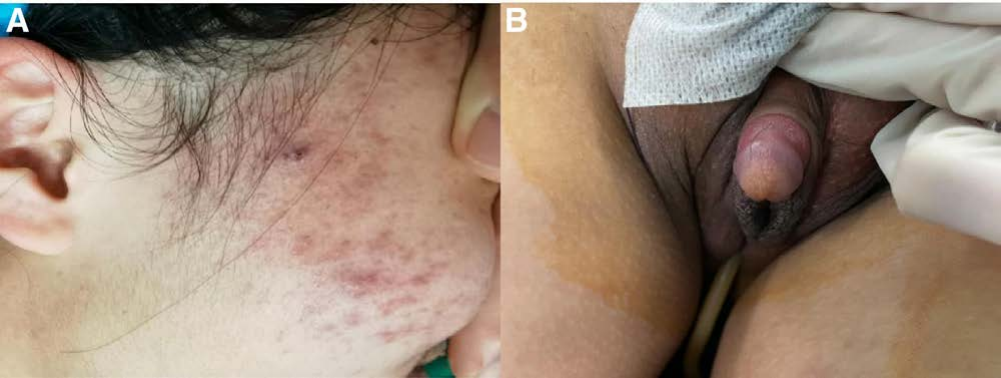
(A) The patient facial hair was exuberant, the skin pores were thick, acne, inflammatory papules, pustules and scars were seen on the face. (B) The patient hypertrophic clitoris.

**Figure 2.**
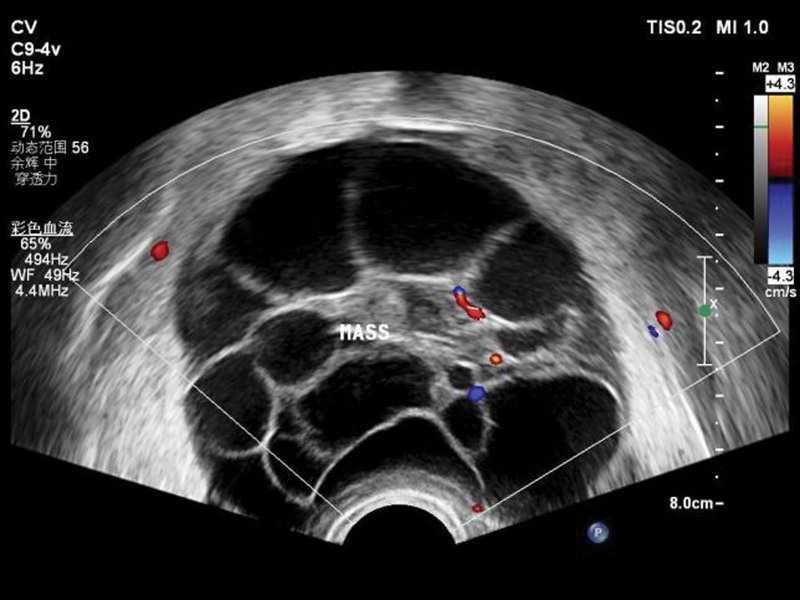
Gynecological ultrasound showed a cystic mass on the right posterior of the uterus, with irregular shape and multiple partitions.

**Figure 3.**
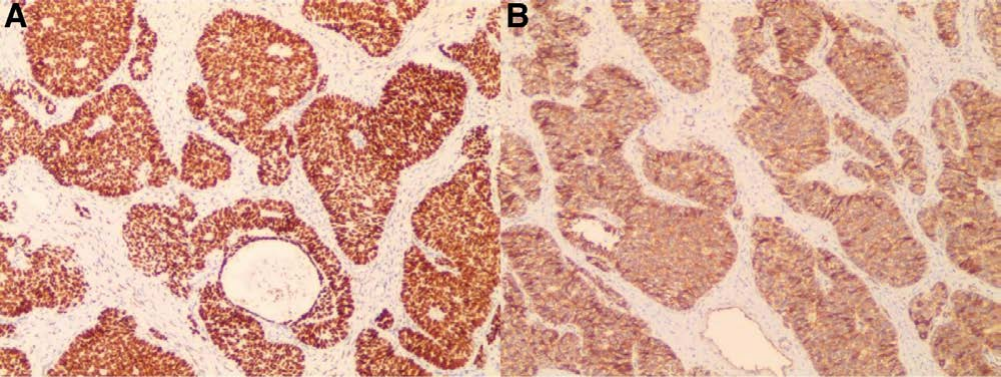
(A) The tumor cells positive for β-Catenin, immunohistochemical staining × 100. (B) The tumor cells positive for FOXL2, immunohistochemical staining × 100.

A 19-year-old patient was hospitalized in the First Affiliated Hospital of Nanjing Medical University due to “amenorrhea for more than 1 year, and discovery of pelvic mass for 4 months” on July 21, 2021. The patient menarche was 14 years old, and her last menstrual period is June 3, 2020. The patient has now had amenorrhea for more than a year and went to the local hospital in March 2021. The gynecological ultrasound showed that the right ovary showed a 75*73*65 mm liquid dark area, separated by light bands, and the capsule was intact. It was expected to diagnose polycystic ovary syndrome in the local hospital. She was given progesterone capsules 100 mg bid for 12 days. The drug was stopped on April 23, 2021, and no withdrawal bleeding has occurred. The patient has severe acne, low voice, and clitoral hypertrophy (Fig. 1) and a mass of about 8 cm in diameter can be reached at the right back of the uterus (Fig. 2). On May 20, 2021, the gynecological ultrasound was performed, and a cystic mass about 88*69*77 mm was seen on the right posterior of the uterus, with irregular shape and multiple partitions. Inside, there was a hyperechoic band that can detect dot-line blood flow signals. Abdominal and pelvic computed tomography (CT) scanning (unenhanced and enhanced) showed a mass with low tissue shadow on the right front of the uterus, with clear boundaries and multiple separations. The larger section was 8*10cm. There was no obvious enhancement in the low density inside but the separations could be strengthened. The right wall of the mass saw the boundary-like soft tissue shadow with mild enhancement during enhancement. The tumor markers (carcinoembryonic antigen/alpha fetoprotein/cancer antigen 125/human epididymis protein 4) show no abnormalities. Follicle-stimulating hormone, luteinizing-hormone, prolactin, progesterone, dehydroepiandrosterone sulfate, human chorionic gonadotropin, anti-Müllerian hormone (AMH) and estradiol are normal, chromosome was 46XX. The concentration of testosterone was 11.36 nmol/L. The initial diagnosis was pelvic mass and secondary amenorrhea. The exploratory laparotomy under general anesthesia on July 23, 2021 showed that about 50 mL of pale-yellow ascites was seen in the pelvic cavity, which was sent for exfoliative cytology. The uterus was slightly smaller, and the right ovary shows cystic enlargement, with a diameter of about 10 cm, with a smooth surface and no adhesion to surrounding tissues. The appearance of the left fallopian tube and ovary and the right fallopian tube were normal, and transabdominal right ovarian tumor resection was performed. The removed right ovarian tumor is sent for rapid pathology, the submitted tissue is multilocular cystic and the sex cord interstitial tumors are not excluded. Routine pathology after surgery is the sex cord tumor. Immunohistochemistry: tumor cells CK-pan (−), Vimentin (−), ER (−), PR (−), P53 (−), SMA (−), CD10 (−), CD34 (−), β-Catenin (+), Inhibin-α (−), CD99 (+), S-100 (−), Syn (−), FOXL2 (+), SF-1 (+), Ki67 (5% +) (Fig. 3), combined with HE Section, this case is consistent with AGCTs. No malignant tumor cells were seen in ascites. The patient was re-hospitalized for laparoscopic right salpingo-oophorectomy and partial omentectomy and biopsy of the peritoneum The testosterone value is quickly down to normal. The patient menstrual cramps on August 13, 2021. Her clitoris was smaller than the front. Up to August 1, 2022, there was no obvious sign of recurrence.

## 3. Discussion

The median age of patients with AGCT at diagnosis is 45 to 63 years old.^[4]^ The patient, in this case, was an adolescent female, and the final diagnosis was AGCT. The main hormones secreted by AGCT are estrogen, inhibin, androgen, and AMH, among which estrogen is the most common.^[3]^ The common symptoms of AGCT are related to the mass itself and the hormones it secretes. If estrogen is secreted, precocious puberty or pubic hair development may occur in childhood, and irregular vaginal bleeding may occur in adolescents or adults.^[5]^ Long-term exposure to high levels of estrogen secreted by AGCT, Some patients may develop endometrial hyperplasia or even endometrial cancer.^[6]^ The Multicenter Italian Trials in Ovarian Cancer Center retrospectively analyzed the data of 150 patients with primary AGCT, and 29.2% of the patients had hyperplasia, while 7.5% of patients develop endometrial cancer.^[7]^ In rare cases, if the tumor secretes androgen, the patient may have hirsutism, clitoromegaly, enlarged abdomen, amenorrhea, deepening of the voice, acne and virilization.^[8]^ Some patients may have abdominal pain due to the rupture of the mass, others include palpable abdominal masses and ascites.^[9]^ But as many as one-fifth of these patients are asymptomatic and are diagnosed accidentally.^[1]^

The tumor size of AGCT is usually between 5 and 15 cm, and more than 95% are unilateral.^[10]^ AGCT is mostly unilateral multilocular cystic solid mass in ultrasound images, and a small part of it is a pure solid or cystic form.^[11]^ Most tumors had a moderate to high color score on doppler evaluation.^[12]^ On CT, AGCTs mainly manifest as cystic solid masses, solid masses and multiple cystic changes. There is no obvious special feature. However, CT allows the abdominal and pelvic cavity to be scanned, to determine whether there is local metastasis, and in the late stage, CT can detect peritoneal metastasis, and at the same time has a certain value for the detection of AGCT recurrence.^[13]^ On magnetic resonance imaging, most AGCTs are solid masses with cystic components or multicystic masses. When the cystic compartments are small and large, the multicystic form can show a sponge-like appearance. This image is characteristic of AGCT. Tumors often have an intra-tumoral hemorrhage; magnetic resonance imaging can reveal the bleeding components of these masses. Since AGCTs are characterized by the production of estradiol, inhibin B, and AMH, these hormones can be used as diagnostic serum markers. The sensitivity of high inhibin B value for a diagnosis of AGCT is 89% and 100%, and the specificity is 91% and 100%. For AMH, the sensitivity varies from 76% to 100%, and the specificity is equivalent to inhibin B.^[14]^ The determination of the serum levels of AMH, inhibin, and estradiol proves that it can also be used to evaluate the effect of treatment and early detection of recurrence.^[15,16]^

Most AGCT with elevated androgen is difficult to distinguish from other androgen-secreting tumors before surgery. The final diagnosis depends on postoperative pathology. In the case of unclear morphology, immunohistochemistry of inhibin α, calmodulin, and FOXL2^[17]^ and mutation of FOXL2 (402C-G)^[18]^ help confirm the diagnosis of AGCT.

The initial surgical treatment of ovarian granulosa cell tumor requires surgical staging according to the ovarian cancer staging system of the International Federation of Gynecology and Obstetrics (FIGO). According to the 2018 European Society of Medical Oncology (ESMO) Guidelines for the Diagnosis and Treatment of Non-epithelial Ovarian Tumors,^[19]^ for women who have completed childbirth, we recommend hysterectomy and bilateral salpingo-oophorectomy. The staging procedure includes omentectomy, a biopsy of the diaphragmatic peritoneum, paracolic gutters, pelvic peritoneum, and peritoneal washings. Generally, systematic lymphadenectomy is not recommended, and only large or suspicious lymph nodes should be removed. According to reports, laparoscopic surgery is safe for stage I patients, but it is necessary to ensure that the tumor does not rupture during the surgery.^[20]^ Because most AGCTs are unilateral, young patients in the local early-stage (stage IA) can choose to preserve the uterus and the contralateral ovary. However, in these cases, careful staging procedures and endometrial sampling should be performed to rule out tumor metastasis and/or concurrent endometrial disease. It is worth noting that there is currently no consensus on whether these patients should undergo radical surgery when they have completed childbirth or reached menopause. For patients who have not undergone a complete staging operation for the first time, laparoscopic re-staging operation is feasible.^[21]^ The relative benefits of adjuvant therapy have not been proven. Stage IA AGCTs have a good prognosis after surgery alone and do not require adjuvant therapy. Compared with IC1 stage, patients in IC2 stage report more recurrences; in this case, adjuvant chemotherapy can be performed, and the most commonly used combination is bleomycin, etoposide and cisplatin (BEP). AGCT is characterized by slow, indolent growth with later recurrence.^[21]^

A long-term follow-up study on AGCTs of the ovary in Taiwan^[5]^ showed that the median time of AGCT recurrence was 57.6 months. Among the recurrence cases, peritoneal recurrence was the most common (77.4%). The total 5-year survival rate and 10-year survival rate of each stage were 96.5% and 94.1%, respectively. Older age, history of previous malignant tumors, higher stage, poor differentiation, larger tumor size, incomplete surgical staging, and residual lesions on surgical margins are independently associated with increased risk of death. These findings highlight the importance of long-term follow-up of patients with AGCT, even those with early-stage disease.

## 4. Conclusion

AGCT is a rare low-grade ovarian tumor. It is rarer to have amenorrhea and hyperandrogenemia as clinical features, and it is easy to be misdiagnosed as polycystic ovary syndrome. Therefore, for patients with abnormally elevated testosterone, clinicians should still be alert to the occurrence of such ovarian tumors. Such patients are prone to relapse many years after the first treatment and require long-term follow-up, which can be monitored by rechecking serum testosterone and pelvic ultrasound.

## Acknowledgments

We thank pathologists of the First Affiliated Hospital with Nanjing Medical University to offer figures and diagnosis the disease.

## Author contributions

**Conceptualization:** Ziwei Jiang, Yujing Li, Xing Chen.

**Data curation:** Ziwei Jiang, Yixuan Qiu, Siwen Hu, Yujing Li.

**Formal analysis:** Ziwei Jiang, Yujing Li, Yichao Jin.

**Supervision:** Xing Chen, Yichao Jin, Huihua Dai.

**Writing – original draft:** Ziwei Jiang, Yujing Li, Xing Chen.

**Writing – review & editing:** Ziwei Jiang, Yixuan Qiu, Siwen Hu, Yujing Li, Xing Chen, Yichao Jin, Huihua Dai.
